# CD40 deficiency mitigates Alzheimer's disease pathology in transgenic mouse models

**DOI:** 10.1186/1742-2094-3-3

**Published:** 2006-02-24

**Authors:** Vincent Laporte, Ghania Ait-Ghezala, Claude-Henry Volmar, Michael Mullan

**Affiliations:** 1 The Roskamp Institute, Sarasota, FL 34243, USA

## Abstract

We have previously shown that transgenic mice carrying a mutant human APP but deficient in CD40L, display a decrease in astrocytosis and microgliosis associated with a lower amount of deposited Aβ. Furthermore, an anti-CD40L treatment causes a diminution of Aβ pathology in the brain and an improved performance in several cognitive tasks in the double transgenic PSAPP mouse model. Although these data suggest a potential role for CD40L in Alzheimer's disease pathology in transgenic mice they do not cast light on whether this effect is due to inhibition of signaling via CD40 or whether it is due to the mitigation of some other unknown role of CD40L. In the present report we have generated APP and PSAPP mouse models with a disrupted CD40 gene and compared the pathological features (such as amyloid burden, astrocytosis and microgliosis that are typical of Alzheimer's disease-like pathology in these transgenic mouse strains) with appropriate controls. We find that all these features are reduced in mouse models deficient for CD40 compared with their littermates where CD40 is present. These data suggest that CD40 signaling is required to allow the full repertoire of AD-like pathology in these mice and that inhibition of the CD40 signaling pathway is a potential therapeutic strategy in Alzheimer's disease.

## Background

The extracellular deposition of the amyloid β-peptide (Aβ) (which is  derived from the processing of the amyloid precursor protein [APP]) in  senile plaques and intracellular accumulation of neurofibrillary tangles  (principally composed of phosphorylated tau protein) are the main  pathological features of Alzheimer's disease (AD) [1]. Besides these lesions, a continuous inflammatory state exists in the brain of AD patients associated with a secretion of pro-inflammatory cytokines around amyloid deposits. The inflammatory response is partly mediated by microglial cells which have been found to be in an activated state in the neighborhood of amyloid cores. It also has been shown that microglia are activated by Aβ exposure *in vitro *[2,3].

It is likely that many factors contribute to regulating the microglial response to Aβ. Previous *in vitro *work shows that Aβ-induced microglial activation is greatly enhanced by stimulation of the CD40 pathway, and that secretion of tumor necrosis factor alpha and neuronal death occur when Aβ-treated microglia are challenged with CD40 ligand, CD40L [3]. Other evidence supports an important role for CD40 and CD40L in AD. For instance, the pattern of expression of these proteins is altered in the brain in AD patients as well as in several animal models of AD [4,5]. In addition, the expression of both APP-processing related genes and genes related to tau-phosphorylation is disturbed in cultured human microglia after treatment with CD40L [6]. Finally, we have shown that mice that express non-functional CD40L and human APP_sw _(APP Swedish, a mutant form of APP that increases the production of Aβ), reduce AD-related pathology such as microgliosis, astrocytosis and Aβ load [7]. Furthermore, an anti-CD40L antibody improves the cognitive function and the AD-related pathology in a double transgenic mouse model (PSAPP) that express human presenilin and human APP_sw _[7,8].

Although a role of CD40L in AD-like pathology in transgenic mice is confirmed by these previous experiments they do not indicate whether CD40 signaling is a necessary requirement for full AD pathology in these models. We thus decided to test whether genetic disruption of CD40 would have a similar effect on the reduction of AD-like pathology as did the removal of functional CD40L. We have generated APP_sw _and PSAPP mouse models deficient for functional CD40 expression and compared the pathological features such as amyloid burden, CD45 and GFAP expression that are typical of AD-like pathology in these transgenic mouse strains with appropriate controls.

## Methods

### Animals

CD40 disrupted mice were purchased from the Jackson Laboratory. This genetic variation occurs on the C57BL/6 background, constructed as described [9]. Tg APP_sw _mice of the 2576 line had a C57B6/SJL background as described [10]. We crossed CD40 disrupted mice with Tg APP_sw _or Tg PSAPP (double transgenic mice with APP_sw _and mutated PS1M146L obtained as described in [11]). The crossed mice obtained will be further referred as: Tg APP_sw_/CD40 deficient (def.) and Tg PSAPP/CD40 def. The offspring were characterized by polymerase chain reaction-based genotyping for mutant APP and PS1 constructs (to determine Tg APP_sw _and PS1M146L status respectively) and for the neomycin selection vector (to type for CD40 deficiency). These mice were then killed at 22 to 24 months of age for pathologic analysis. Littermates were used as controls throughout. Animals were given food and water *ad libitum*. They were housed and maintained in the Roskamp Institute Animal Facility, and all experiments were in compliance with protocols approved by the Roskamp Institute Institutional Animal Care and Use Committee.

### Brain preparation

Mice were anesthetized with isoflurane, then the brains (Control, Tg APP_sw_, CD40 def., TgAPP_sw_/CD40 def., PSAPP/CD40 def. and PSAPP) were isolated under sterile conditions on ice. One hemisphere of each brain was immersed in 4% paraformaldehyde at 4°C overnight, and routinely processed in paraffin. Briefly, the hemispheres were embedded into paraffin using Tissue-Tek (Sakura, USA) and sagitally cut into 6-um-thick sections with a microtome (2030 Biocut, Reichert/Leica, Germany). Sections were mounted on slides, air-dried and stored until needed. The second hemisphere was placed in ice-cold lysis buffer (20 mM Tris, pH 7.5, 150 mM NaCl, 1 mM EDTA, 1 mM EGTA, 1% v/v Triton X-100, 2.5 mM sodium pyrophosphate, 1 mM β-glycerolphosphate, 1 mM Na_3_VO_4_, 1 μg/mL leupeptin, 1 mM PMSF), then sonicated on ice for approximately 3 min, let stand for 15 min at 4°C, and centrifuged at 15,000 rpm for 15 min.

### Total Aβ extraction and quantification

Total Aβ species were detected by acid extraction of brain homogenates. Insoluble Aβ species were extracted by 70% formic acid as previously published [12]. Aβ content in brain was determined using human Aβ 1–40 and Aβ 1–42 ELISA kits (BioSource, Camarillo, CA) in accordance with the manufacturer's instruction. Data are expressed as pg/mg protein, mean ± s.e.m.

### Antibodies

Monoclonal antibody AT8 (Pierce Biotechnology, IL) which recognizes human phosphorylated tau at Ser202 and Thr205 was diluted 1:400. CD45 was immunodetected using a 1:50-dilution of a rat monoclonal antibody (clone IBL-3/16) from Serotec, NC. Rabbit anti-cow glial fibrillary acidic protein (GFAP) was used at 1:1000 (Dakocytomation, CA). Monoclonal antibody 4G8 was used to stain Aβ deposits at a 1:750-dilution and purchased from Signet Laboratories, MA. Rabbit anti-Aβ 1–40 and rabbit anti-Aβ 1–42 were used at 1:100 and provided by Chemicon, CA. F4/80 antigen was detected using a rat antibody (clone CI:A3-1 from Serotec) diluted 1:50.

### Immunohistochemistry and Congo red staining

Prior to staining, sections were deparaffinized in xylene (2 × 5 min) and hydrated in graded ethanol (2 × 5 min in 100%, 5 min in 85%, 5 min 70%) to water.

Endogeneous peroxidase activity was quenched with a 20-min-H_2_O_2 _treatment (0.3% in water) and after being rinsed, sections were incubated with blocking buffer (Protein Block Serum-free, DakoCytomation) for 20 min. The diluted antibodies were applied onto the sections overnight at 4°C. They were detected using the Vectastain ABC (avidin-biotin-peroxidase complex) Elite kit (Vector Laboratories, CA) and the labeling was revealed by incubating sections in 0.05 M Tris-HCl buffer (pH 7.6), containing 3,3'-diaminobenzidine (Sigma, MO) and H_2_O_2_.

Congo red staining was performed using the kit from Sigma according to the manufacturers' indications.

For each brain, 4 to 5 stained sections were used to perform the quantification. The stained area within particular regions (hippocampus, cortex or  subfields of cortex) was quantified using the Image-Pro Plus software (Media  Cybernetics, MD). An average value was calculated for each area from individual mice. These averages were used to estimate the overall staining for each genotype. The relationships between staining in each brain area and genotype were examined by one-way analysis of variance and post hoc test.

## Results

**Figure 1:**
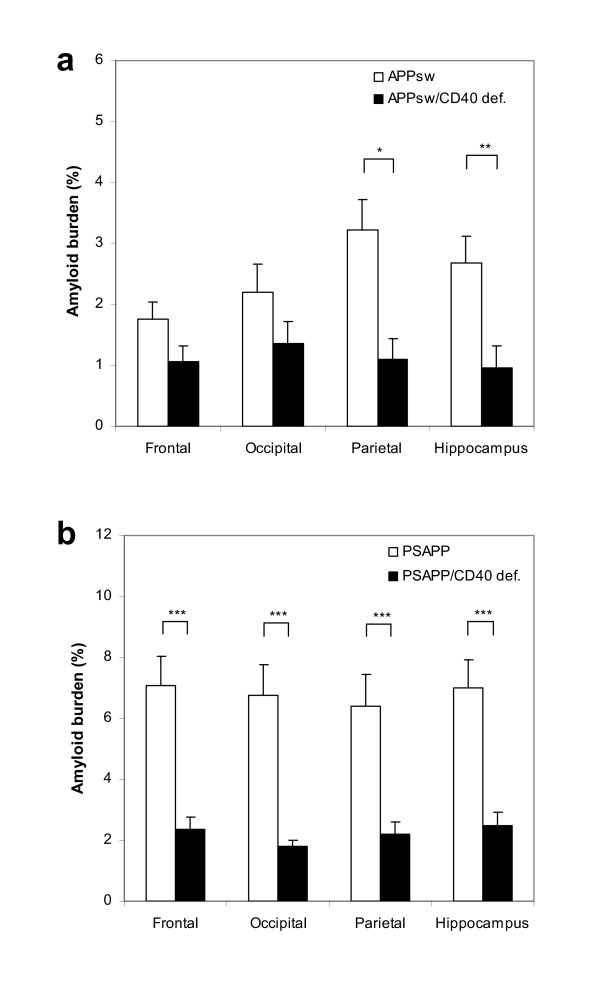
Percentages of 4G8-positive β-amyloid plaques (mean ± s.e.m) by area in (a) Tg APP_sw_/CD40 def. mice versus Tg APP_sw _mice and in (b) Tg PSAPP/CD40 def. mice versus Tg PSAPP mice at 22 to 24 months of age calculated by quantitative image analysis. Post hoc comparison between groups are indicated by the marked bars (* *p *< 0.05 ; ** *p *< 0.01 ; *** *p *< 0.001).

**Figure 2:**
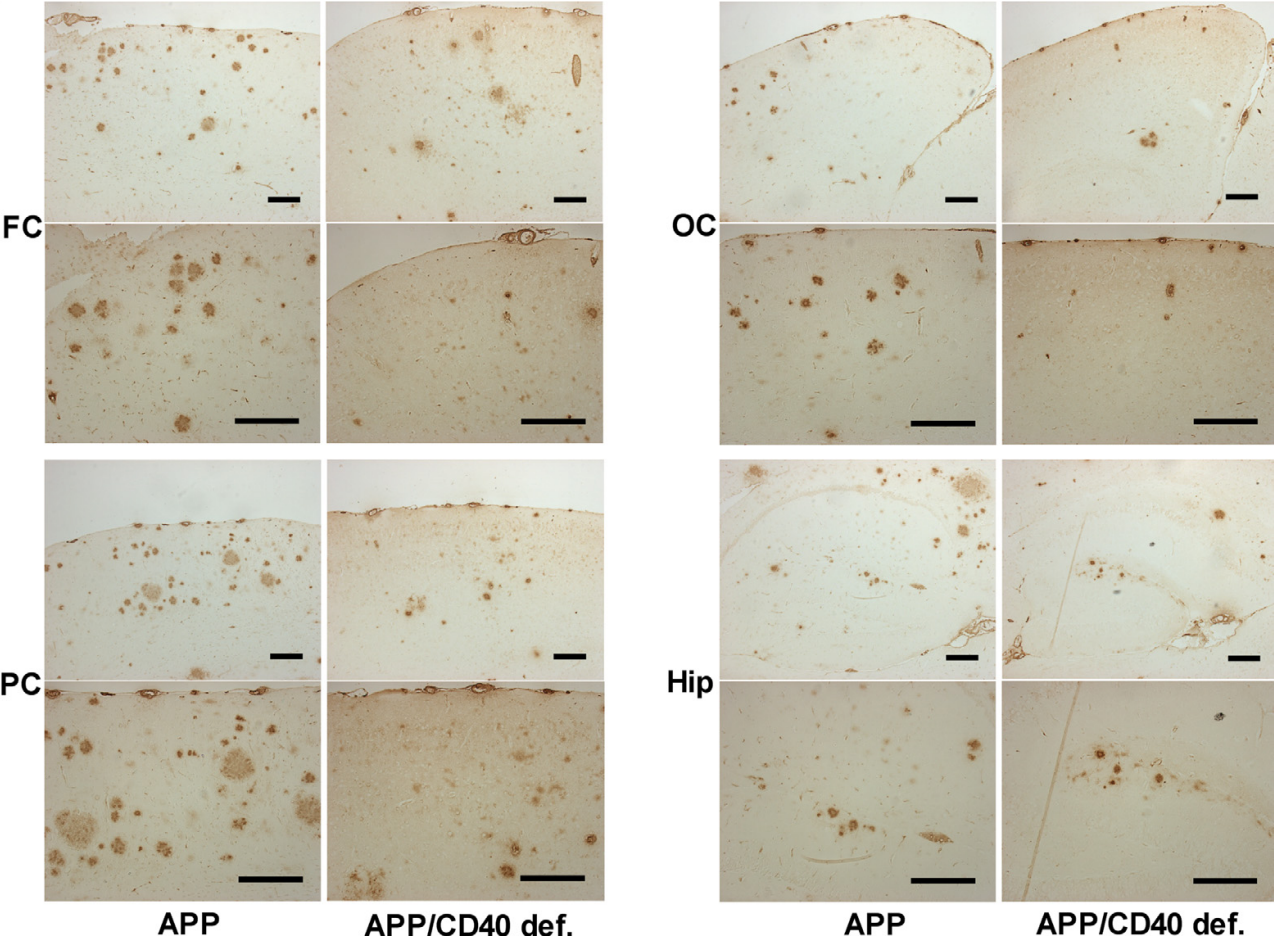
Representative photographs of frontal, occipital and parietal cortical areas and hippocampus in Tg APP_sw _and Tg APP_sw_/CD40 def. stained with 4G8 antibody (each bar represents 0.2 mm; FC : frontal cortex; OC : occipital cortex; PC : parietal cortex; Hip: hippocampus).

**Figure 3:**
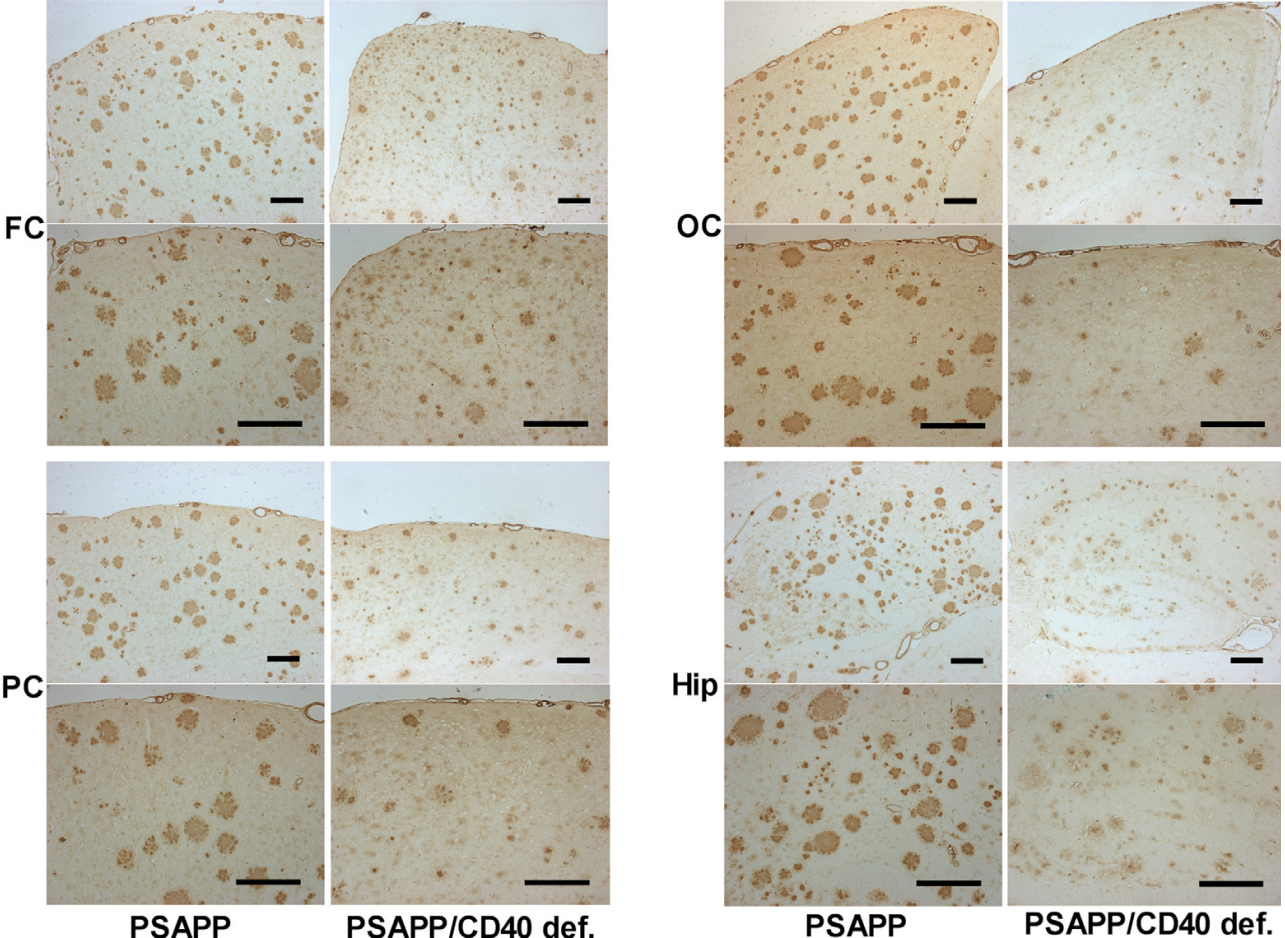
Representative photographs of frontal, occipital and parietal cortical areas and hippocampus in Tg PSAPP and Tg PSAPP/CD40 def. stained with 4G8 antibody (each bar represents 0.2 mm; FC : frontal cortex; OC : occipital cortex; PC : parietal cortex; Hip: hippocampus).

Both Tg APP_sw_/CD40 def. and Tg PSAPP/CD40 def. show decrease in 4G8-positive plaques compared to their Tg APP_sw _and Tg PSAPP littermates. There is a reduction in brain area specific amyloid load in the Tg APP_sw_/CD40 def. mice by 38% (occipital cortex) to 66% (parietal cortex) (Table 1). Similarly, the PSAPP/CD40 def. mice show 65% (hippocampus) to 73% (occipital cortex) reduction compared to their Tg APP_sw _and Tg PSAPP littermates. In a post hoc comparison of the means, only the differences in the parietal cortex and hippocampus were significant in the Tg APP_sw _vs Tg APP_sw_/CD40 def. mice (Fig. 1a and Fig. 2). By contrast, all brain areas showed significant post hoc differences for the PSAPP vs PSAPP/CD40 def. mice (Fig. 1b and Fig. 3).

**Table 1:** Value of 4G8-positive β-amyloid plaques and percentage of reduction when CD40 is disrupted in Tg APP_sw _and Tg PSAPP mice.

**Genotype **Studied area	4G8-positive plaque (% of area)	Variation (%) when compared to control
**Tg APP>_sw_**		
Frontal cortex	1.75 ± 0.29	n/a*
Occipital cortex	2.20 ± 0.46	n/a
Parietal cortex	3.22 ± 0.48	n/a
Hippocampus	2.67 ± 0.45	n/a
**Tg APP**_sw_**/CD40 def.**		
Frontal cortex	1.06 ± 0.24	40% reduction
Occipital cortex	1.37 ± 0.33	38% reduction
Parietal cortex	1.11 ± 0.32	66% reduction
Hippocampus	0.97 ± 0.30	63% reduction
**Tg PSAPP**		
Frontal cortex	7.06 ± 0.96	n/a
Occipital cortex	6.77 ± 1.01	n/a
Parietal cortex	6.39 ± 1.03	n/a
Hippocampus	6.99 ± 0.93	n/a
**Tg PSAPP/CD40 def.**		
Frontal cortex	2.34 ± 0.41	67% reduction
Occipital cortex	1.80 ± 0.19	73% reduction
Parietal cortex	2.19 ± 0.39	66% reduction
Hippocampus	2.47 ± 0.43	65% reduction

*non applicable

**Figure 4:**
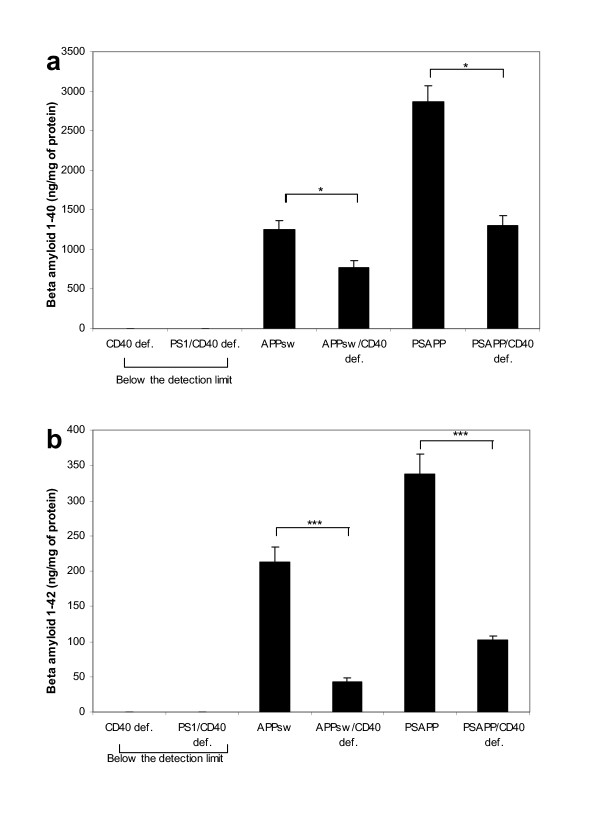
Formic-extracted (a) Aβ_1–40 _and (b) Aβ_1–42 _(mean ± s.e.m) measured by ELISA in Tg APP_sw_, Tg PSAPP, Tg APP_sw_/CD40 def. and Tg PSAPP/CD40 def. Post hoc analysis between groups are indicated by the marked bars (* *p *< 0.05; *** *p *< 0.001).

As shown in Fig. 4a and 4b, ELISA analysis of formic-extracted Aβ produced results consistent with the above findings (mean Aβ, pg/mg of protein brain ± s.e.m., Tg APP_sw _mice vs Tg APP_sw_/CD40 def.; 30% reduction in Aβ_1–40 _and 80% in Aβ_1–42 _[1,249,460 ± 110,868.31 vs 728,181.33 ± 142,274.44 and 212,631.87 ± 21,453.03 vs 42,266.92 ± 5,782.35 respectively]. Tg PSAPP mice vs Tg PSAPP/CD40 def.; 45% reduction in Aβ_1–40 _and 70% reduction in Aβ_1–42 _[2,868,709.33 ± 198,843.63 vs 1,298,021.33 ± 133,263.81 and 337,169.33 ± 28,240.97 vs 102,307.33 ± 5,363 respectively]).

**Figure 5:**
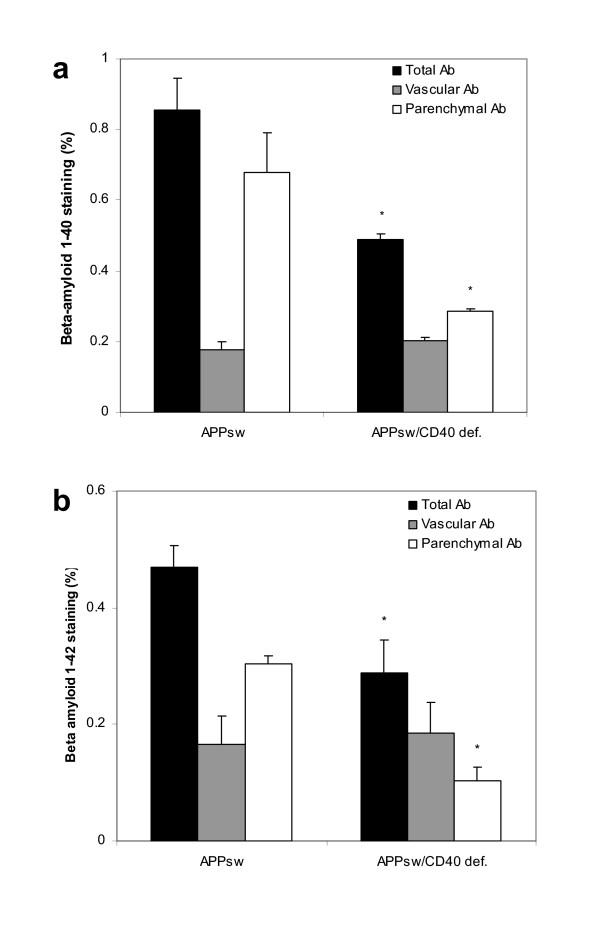
Total, vascular or parenchymal-deposited (a) Aβ_1–40 _and (b) Aβ_1–42 _expressed as a percentage (mean ± s.e.m) of the area studied in Tg APP_sw_/CD40 def. mice versus Tg APP_sw _mice at 22 to 24 months of age calculated by quantitative image analysis. Post hoc comparison between groups are indicated by the marked bars (* *p *< 0.05).

Staining of Tg APPsw and Tg APPsw/CD40 def. brain with specific antibodies directed against Aβ_1–40 _and Aβ_1–42 _and analysis of their parenchymal or vascular localization have been performed. As expected, the overall immunostaining for Aβ_1–40 _and Aβ_1–42 _was reduced by 43% and 38% respectively, in Tg APPsw/CD40 def. mice when compared to Tg APPsw. However, an analysis of the parenchymal and vascular deposits of each Aβ species showed that only the parenchymal-deposited Aβ_1–40 _and Aβ_1–42 _was reduced when CD40 is disrupted, whereas the vascular-associated Aβ is essentially unchanged (Fig. 5a and 5b). Consequently, the vascular Aβ_1–40 _represented 20% of the total Aβ_1–40 _in Tg APP_sw_, whereas it represented 40% in Tg APP_sw_/CD40 def. The values are 36% and 64% respectively for vascular Aβ_1–42_.

**Figure 6:**
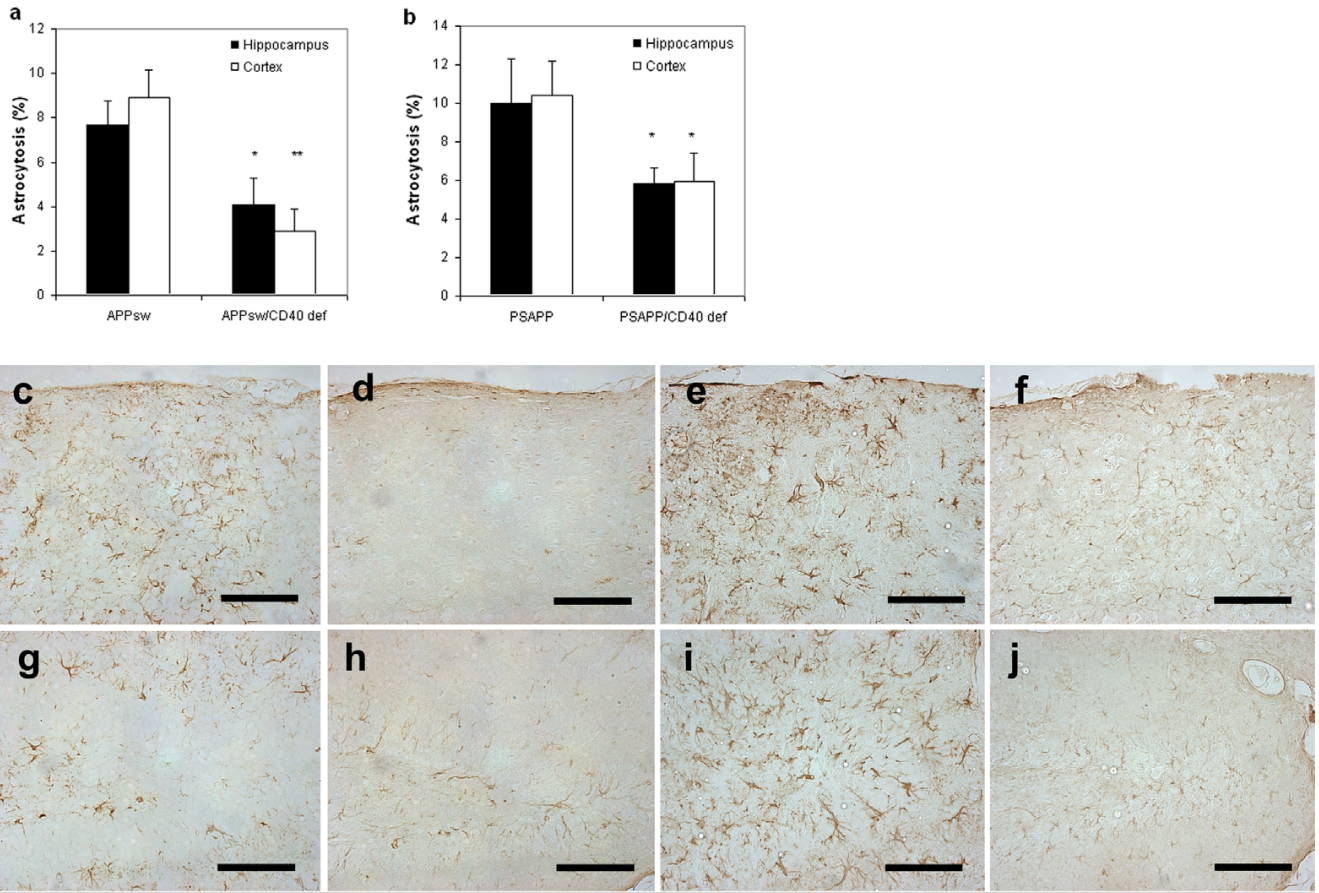
Percentage of astrocytosis (mean ± s.e.m) by area in (a) Tg APP_sw_/CD40 def. mice versus Tg APP_sw _mice and in (b) Tg PSAPP/CD40 def. mice versus Tg PSAPP mice at 22 to 24 months of age calculated by quantitative image analysis. Post hoc comparison between groups are indicated by the marked bars (* *p *< 0.05; **p < 0.01). Representative photographs of (c, d, e, f) cortex and (g, h, i, j)  hippocampus in (c, g) Tg APP_sw_, (d, h) Tg APP_sw_/CD40 def., (e, i) Tg PSAPP  and (f, j) Tg PSAPP/CD40 def. stained with GFAP antibody (each bar  represents 0.1 mm).

The disruption of the CD40 gene in Tg APP_sw _and in Tg PSAPP mice leads to a decrease of reactive astrocytes quantified by GFAP staining and image analysis (34% reduction in the hippocampus and 53% reduction in the cortex of Tg APP_sw_/CD40 def.; 32% reduction in the hippocampus and 33% reduction in the cortex of Tg PSAPP/CD40 def.) (Fig. 6).

**Figure 7:**
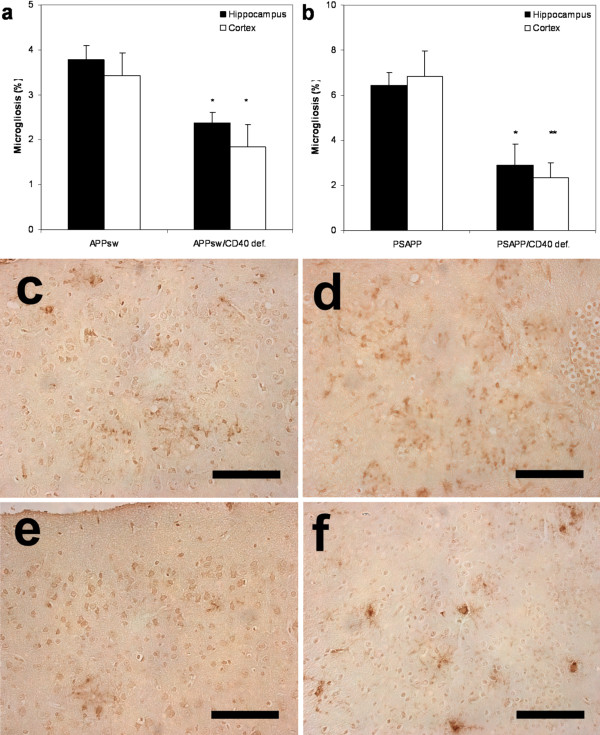
Percentage of microgliosis (mean ± s.e.m) by area in (a) Tg APP_sw_/CD40 def. mice versus Tg APP_sw _mice and in (b) Tg PSAPP/CD40 def. mice versus Tg PSAPP mice at 22 to 24 months of age calculated by quantitative image analysis. Post hoc comparison between groups are indicated by the marked bars (* *p *< 0.05; ** *p *< 0.01). Representative photographs of brain area in (c) Tg APP_sw_, (e) Tg APP_sw_/CD40 def., (d) Tg PSAPP and (f) Tg PSAPP/CD40 def. mice stained with CD45 antibody (each bar represents 0.1 mm).

There was a concomitant reduction in CD45-stained microglia which was reduced by 37% in the hippocampus and 46% in the cortex when CD40 is disrupted in Tg APP_sw _(Fig. 7a, c and 7e). The reduction reached 55% (hippocampus) and 65% (cortex) when PSAPP/CD40 def. and PSAPP mice are compared (Fig. 7b, d and 7f).

**Figure 8:**
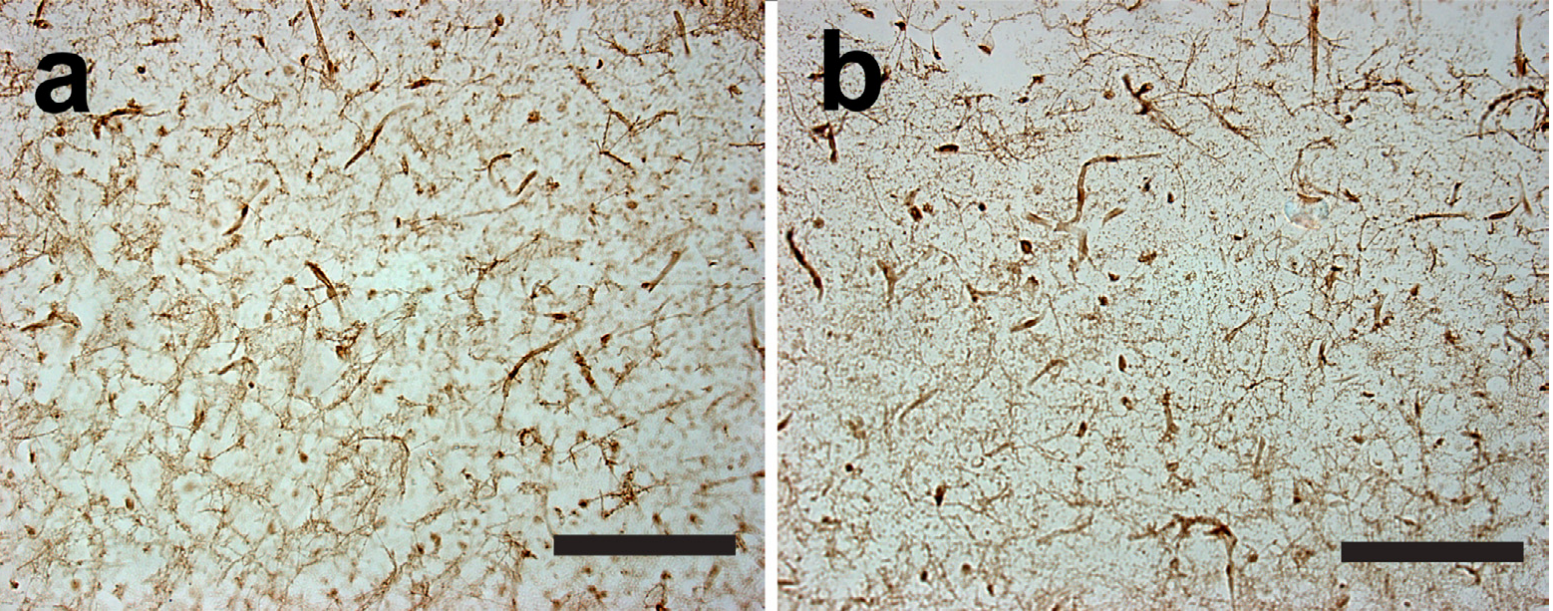
Representative photographs of cortical area in (a) CD40 def. and (b) wild-type mice stained with F4/80 antibody at 8 to 10 weeks old (each bar represents 0.1 mm).

Since CD40 deficiency could impair the development or differentiation of microglia in mice, we have stained brain sections of 8 to 10 weeks old mice for F4/80 antigen. As shown in Fig. 8, no difference was observed between CD40 def. mice and wild type mice. Quantification of the stained area confirmed the observation: 15.68 ± 1.23 % and 12.51 ± 1.76 % of the cortex were stained for F4/80 in CD40 def. mice and wild type mice respectively.

It has been shown that congophilic plaques are associated with phosphorylated tau-immunoreactive aberrant structures in Tg APP_sw _[13]. Therefore, we explored the presence of these structures using an anti-phosphorylated-tau antibody AT8 (tau phosphorylated at Ser202 and Thr205) in the transgenic mice. These structures were exclusively associated with congophilic plaques and were not found in the control mice. The mean ratio of areas of phosphorylated tau to Congo red in Tg APP_sw _is unchanged with age (from 11 to 20.5 months old) and is approximately 10% as previously reported [13]. We have found a similar ratio in these mice at 22 to 24 months old and the genetic deletion of CD40 did not disrupt this ratio in either Tg APP_sw _or Tg PSAPP mice. However, as the total amyloid burden was reduced in the CD40 def. animals, overall there was a concomitant reduction in AT8-positive staining (data not shown).

## Discussion

It has previously been shown that reduction of functional CD40L mitigates AD like-pathology in transgenic mouse models of AD [3,7]. The mechanism of this effect is not known but an obvious possibility is that CD40L promotes AD-like pathology by activating CD40. Other possibilities include a direct role of CD40L in Aβ fibrillogenesis or oligomer formation. In order to test whether CD40 transduction is needed to promote AD-like pathology in these transgenic mouse models, we examined transgenic mice with and without functional CD40. All the pathological evidence suggests that CD40 itself is required to promote amyloid, tau and glial pathologies.

The mitigation of all these pathologies is greatest in the transgenic animals with most amyloid over-expression. This observation may be due to the fact that more accurate quantification is possible when pathologies are denser or it may truly reflect a rate limiting step imposed by CD40.

We have recently shown in a genomic study that CD40 ligation of microglia specifically up-regulates APP metabolism and tau phosphorylation related genes [6]. Although microglia do not produce Aβ, it is possible that in neurons (where CD40 is expressed [14]) CD40 ligation may enhance Aβ production. This may be also true in astrocytes [15,16].

Additionally, the activation of microglia by CD40L may enhance the inflammatory response in these mouse models and promote Aβ production from neurons. For instance CD40L-stimulated microglia release IL1β and α which increase APP expression and IL1β increases APP metabolism to Aβ [17,18].

Thus, in the presence of a fully functional CD40 transduction and Aβ, microglia may be highly activated (compared to their response when CD40 transduction can not take place [3]) and in turn, may induce further Aβ production via neuro-inflammation. It is interesting to note that the presence of CD40 promotes parenchymal deposition of Aβ but does not impact vascular deposits. It maybe that CD40 only impacts production of Aβ from parenchymal but not vascular cells. Alternatively, it is possible that CD40 impairs the transfer of Aβ across the blood-brain barrier and that its deficiency facilitates the transport of parenchymal Aβ to the periphery. Disproportionate accumulation of Aβ in or around the vasculature might occur under these circumstances.

The downstream signaling events in the CD40 pathway which lead to Aβ production and tau phosphorylation need further exploration. In particular, it will be important to separate NF-κ b-induced pro-inflammatory responses known to occur in microglia from other NF-κ b signaling directly related to APP processing. As Aβ itself can activate NF-κ b (which is enhanced in AD brain) both activated CD40 and Aβ may synergistically enhance NF-κ b signaling resulting in feed-forward Aβ production with the consequent cascade of other AD pathologies.

## List of abbreviations

Aβ (amyloid β-peptide); AD (Alzheimer's disease); APP (amyloid precursor protein); def. (deficient); GFAP (glial fibrillary acidic protein); PS (presenilin); Tg (transgenic).

## Competing interests

The author(s) declare that they have no competing interests.

## Authors' contributions

VL carried out the immunohistochemistry studies and drafted the manuscript. GAG conceived the study and its design, and helped to draft the manuscript. CHV carried out the immunoassays. MM has been involved in drafting the manuscript. All authors read and approved the final manuscript.
